# Do Individuals’ Activity Structures Influence Their PM_2__.__5_ Exposure Levels? Evidence from Human Trajectory Data in Wuhan City

**DOI:** 10.3390/ijerph18094583

**Published:** 2021-04-26

**Authors:** Siyu Ma, Lin Yang, Mei-Po Kwan, Zejun Zuo, Haoyue Qian, Minghao Li

**Affiliations:** 1 School of Geography and Information Engineering, China University of Geosciences, 388 Lumo Road, Wuhan 430074, China; masiyu@cug.edu.cn (S.M.); zjzuo@cug.edu.cn (Z.Z.); qhy_2015@cug.edu.cn (H.Q.); liminghao@cug.edu.cn (M.L.); 2 National Engineering Research Center of Geographic Information System, China University of Geosciences, 388 Lumo Road, Wuhan 430074, China; 3 Department of Geography and Resource Management, Institute of Space and Earth Information Science, The Chinese University of Hong Kong, Shatin, Hong Kong, China; mpkwan@cuhk.edu.hk; 4 Department of Human Geography and Spatial Planning, Utrecht University, 3584 CB Utrecht, The Netherlands

**Keywords:** PM_2__.__5_ exposure, human mobility, cell phone GPS dataset, activity patterns, PM_2__.__5_

## Abstract

Severe air pollution has become a major risk to human health from a global environmental perspective. It has been recognized that human mobility is an essential component in individual exposure assessment. Activity structure reflects the characteristics of human mobility. Thus, a better understanding of the relationship between human activity structure and individual exposure level is of crucial relevance. This study examines this relationship using a large cell-phone GPS dataset in Wuhan, China. The results indicate that there is a strong linear relationship between people’s activity structures and exposures to PM_2__.__5_. Inter-group comparisons based on the four activity structure groups obtained with K-means clustering found that groups with different activity structures do experience different levels of PM_2__.__5_ exposure. Furthermore, differences in detailed characteristics of activity structure were also found at different exposure levels at the intra-group level. These results show that people’s activity structures do influence their exposure levels. The paper provides a new perspective for understanding individual exposure through human activity structure, which helps move the perspective of research on individual exposure from the semantic of physical location to the semantic of human activity pattern.

## 1. Introduction

Severe air pollution has become a global environmental problem and a major risk to human health [1,2,3,4]. As the world’s largest developing country, China has experienced an unprecedented urbanization process and rapid economic growth in the past 30 years, with an average annual growth in GDP of about 9%. Meanwhile, the country’s air pollution problem adversely affects the health of its population. Air pollution can lead to a variety of health problems, such as respiratory and cardiovascular issues, lung cancer, and even premature death [5,6]. It has become a major public health concern in China. As a result, more and more studies in recent years focus on the assessment of people’s exposure to and the health impact of air pollution [7,8,9].

Health geographers and public health researchers have conducted pollution exposure assessments for decades. Traditionally, residence-based measurement, where the residential neighborhood is used as the contextual area, has been largely used in exposure assessment. However, this method ignores the impact of individuals’ mobility on their exposure to environmental risk factors and may lead to the neighborhood effect averaging problem (NEAP) [10,11]. Most people undertake their daily activities outside their residential neighborhoods at different places. Thus, ignoring people’s daily mobility and exposures to nonresidential contexts may lead to erroneous results in studies of exposure estimates [6,12,13,14,15,16]. For accurate assessment of the health effects of pollution exposure, the changes in individual daily mobility and activity-travel microenvironments need to be included to obtain individual-level exposure at fine spatiotemporal scales. For instance, differences in personal exposures to fine particulates associated with different activity places (e.g., homes, workplaces, shops and outdoor locations) have been examined [9,17,18] and differences between real-time sensing and static monitoring station-based estimates have also been revealed. In addition, the variation in exposure estimates for multiple travel modes (such as cars, bicycles or motorcycles, public transport and walking) across various environmental conditions and the variations on days with low, middle and high air pollution levels have also been observed [3,19]. Related research findings indicate that air pollution concentrations vary substantially across various environmental conditions. These findings help to improve our understanding of individual exposure to air pollution from a static residential-based point of view to a dynamic mobility-based perspective.

Human mobility is widely recognized as a necessary component in people’s exposure assessment since people organize their lives in different places in the city [10]. Conceptually, human mobility describes how individuals move within a network or system. Thus, knowing when and where people undertake activities and how these activities are spatially and temporally structured is important for capturing the characteristics of human mobility. It can reveal the characteristics of the individuals conducting different activities over time and reflect variations in individual daily activities (how they change over time). The daily activities of groups of individuals in cities tend to have underlying structures (regular yet rich dynamics in their social and physical lives). In addition, it has been demonstrated that exposure levels are often different when people are exposed to different environments or activity contexts. Indeed, human activities influence the timing, location, and degree of pollutant exposure and play a key role in examining exposure variation [20]. Thus, in the context of different daily activity structures, human trajectories can represent people’s movements between places with different air exposure levels and can be used to examine how individuals may experience different levels of exposure to air pollution. Because the link between people’s daily activity structures and exposure levels is not self-evident, our understanding of the associations between human activity structure and individual exposure is still limited to date. Thus, a better understanding between them is of crucial relevance. This study seeks to investigate the relationship between people’s daily activity structure and their exposure levels and whether exposure levels can be interpreted in terms of activity structure semantics.

During the last two decades, although the assessment of people’s exposure to environmental risks has evolved from a static residential-based point of view to a dynamic mobility-based perspective, past studies are still largely location-based. The research community mostly focuses on measuring the exposure in specific activity contexts or specific microenvironments. Generally, human activity is interpreted as a description of a combination of sequences of activities that occur over a longer period of time. However, the implicit semantics of this structure is essential to reflect the characteristics of human mobility. Further, most existing studies are based on location-based observations. The measurement and interpretation of exposure need to go beyond the meaning of the physical location to the semantics of the human activity pattern. However, researches on the interpretation of exposure from the perspective of daily activity structure are still limited.

From the perspective of the health impact of air pollution, health disparities across socioeconomic groups have been widely studied from the perspective of socio-economic attributes [2]. Sample survey data or longitudinal observation data have been used to explore the health effects of various environmental factors on different social groups, such as low-income people, African Americans, children, pregnant women and other disadvantaged groups [21,22,23]. These studies are usually based on the socio-economic attributes of the sample population obtained through questionnaires to examine the exposure differences between different social groups. Time-activity information is usually included to obtain an accurate individual exposure assessment. However, few studies measure the linkage between human activity structure and the associated exposure results in a quantitative manner, and the impact of human activity structure on individual exposure to air pollution has not been well investigated or understood.

The investigation of the relationship between people’s daily activity structure and their exposure levels has now become possible due to the well-developed methods of identifying activity patterns and behaviors in the field of geographic information science (GIScience) and computer science communities [24,25,26,27,28] and the availability of human trajectory data. Therefore, the main objective of this study is to better understand the relationship between the daily activity structure and the air pollution exposure level (taking PM_2__.__5_ as an example) of city dwellers. Using the mobile phone GPS data of 15,120 users on a weekday in Wuhan, China, this paper aims to examine two issues: (1) Whether individuals’ activity structures influence their exposure level to PM_2__.__5_; (2) If it is the case, how different daily activity structures potentially affect the PM_2__.__5_ exposure levels people experienced at the group level? To address these questions, the study will first examine the associations between individual activity structure and exposure to PM_2__.__5_ using coefficient analysis and linear regression. Then, we group individuals according to their similar activity structures and investigate the interactions between activity structure and exposure level at the inter- and intra-group levels. The results will reveal whether activity structure has a significant impact on the exposure level of our sample population. The findings will enhance our understanding of how different activity structures affect individuals’ exposure to air pollution.

## 2. Study Area and Data

**Figure 1:**
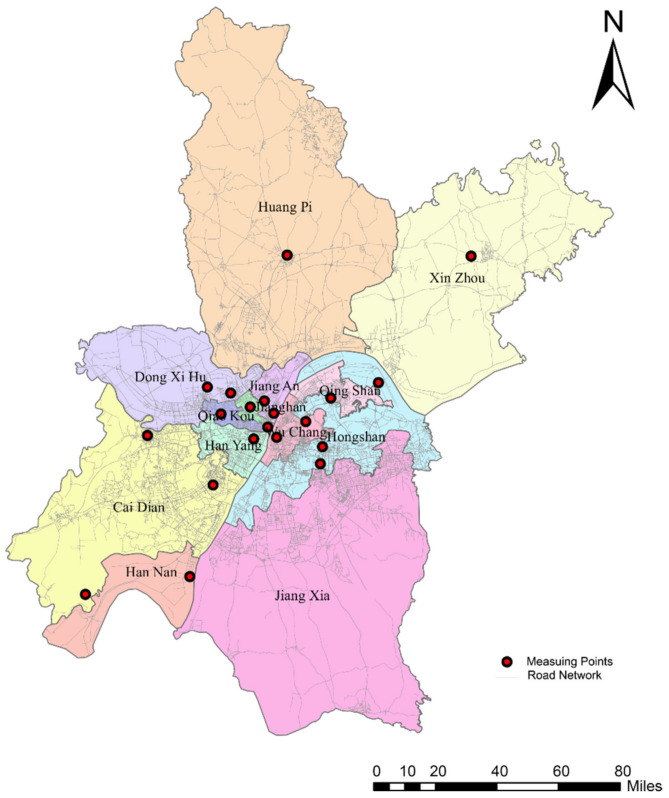
Administrative Districts of Wuhan city, China.

The study area for this research is Wuhan, which is the capital and largest city of the Chinese province of Hubei in central China. It includes seven central districts (Jiang’an, Jianghan, Qiaokou, Hanyang, Wuchang, Qingshan, and Hongshan) and six outer districts (Dongxihu, Hannan, Caidian, Jiangxia, Huangpi, and Xinzhou). Figure 1 shows the administrative area of Wuhan, which has an area of 8569.15 square kilometers and a population of about 11.21 million. The data of the administrative districts used in this paper is from the Hubei Province Bureau of Surveying and Mapping Geographic Information and the road network data is from OpenStreetMap (OSM), which is a free spatial data source based on crowdsourced data.

The location dataset used in this study is from the phone GPS data of the largest Chinese food delivery platform—Meituan (www.meituan.com, accessed on 11 March 2019). Its daily active users have reached 69.85 million by December 2019. The data mainly includes the location (i.e., user ID, longitude coordinate, latitude coordinate, and recording time) of 1,141,446 users of the platform on 11 March 2019. The location data were recorded only when the GPS location request was allowed. Therefore, the raw data have been cleaned to make sure that the location records of each selected individual user have at least one location record every 30 min from 6:00 a.m. to 24:00 p.m. Finally, 450,950 location records were selected from 15,120 individuals as our location dataset.

In the study area, there are 10 national-level and 12 municipal-level air quality monitoring stations run by the Wuhan Ecology and Environment Bureau. The locations of these 22 monitoring stations are represented as red dots in Figure 1. The pollutants monitored include sulfur dioxide (SO_2_), nitrogen dioxide (NO_2_), ozone (O_3_), carbon monoxide (CO), particulate matter PM_2__.__5_ (particle size smaller than or equal to 2.5 μm) and particulate matter PM_10_ (particle size smaller than or equal to 10 μm). The air quality data of each monitoring station is published every hour. Since PM_2__.__5_ is the main typical pollutant and has great harm to the human respiratory system, we choose it as the representative pollutant to evaluate personal exposure to air pollution. Therefore, the PM_2__.__5_ data were collected from a total of 22 observation points on the same day as the location dataset. The PM_2__.__5_ concentrations on 11 March 2019, ranged from the minimum value of 51.93 μg/m^3^ to the maximum value of 83.41 μg/m^3^, with an average value of 67.67 μg/m^3^. The air quality is placed in the light pollution category. 

## 3. Methodology

### 3.1. Data Preparation

Since there are missing data in the original GPS trajectory data (e.g., the location information is not recorded at certain moments for some users), the original dataset needs to be filtered. 30 min is chosen as an interval and a total of 37 time points are set between 6:00 a.m. and 24:00 p.m. If the original trajectory data of a user has geographic location information at all these time points, then the location coordinates at these time points are orderly constructed to form the trajectory data of the user. A trajectory data set is constructed after the filtering process. Each individual’s trajectory is defined as:

(1) $$tr=\left\{\left(long_{1},lat_{1}t_{1}\right),\ldots \ldots ,\left(long_{i},lat_{i},t_{i}\right),\ldots \ldots ,\left(long_{n},lat_{n},t_{n}\right)\right\}$$

where (*long_i_*, *lat_i_*, *t_i_*) represents the individual’s longitude and latitude coordinates at time point *t_i_* and *n* represents the total number of time points.

Then, among the six outer suburban districts in Wuhan, Huangpi, Xinzhou and Jiangxia districts cover a relatively large area (Huangpi district with 2256.7 km^2^, Xinzhou district with 1500.66 km^2^, and Jiangxia district with 2018.3 km^2^) and with only one or no monitoring station. Thus, the users whose trajectories passed these areas are removed to mitigate the effect of the limited number of air quality monitoring stations in these districts. Finally, 15,120 users are selected as the sample dataset.

### 3.2. Representation of Individual Activity Structure

**Figure 2:**
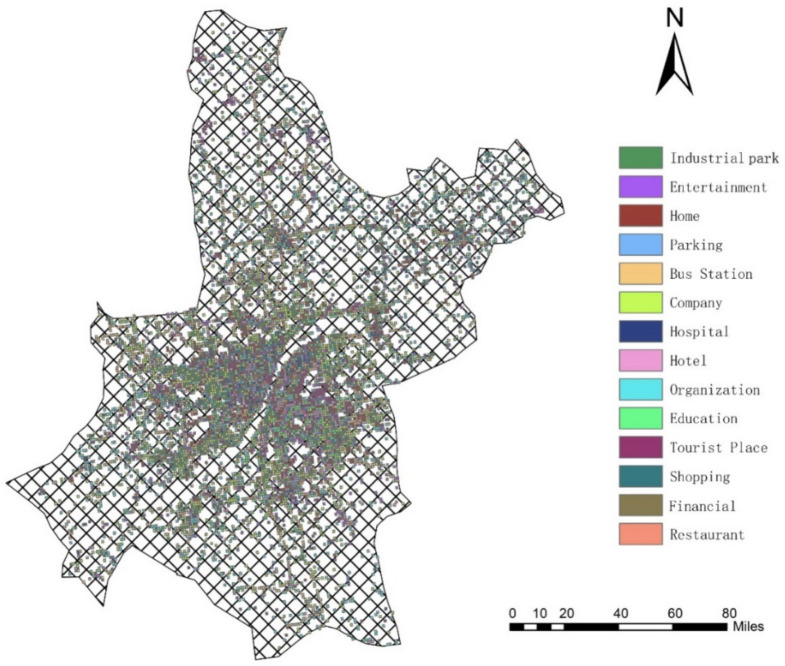
POI Classification of Wuhan in the unit of 100 × 100 one-meter grid.

To better understand the behavioral pattern of moving objects and create more accurate models, Spaccapietra et al. [29] introduced the semantic trajectories where the human semantic trajectory is defined generally as a sequence of semantical locations (e.g., “school”, “restaurant”, and so on). In this study, the notion of activity structure captures individual activity variations at different times of the day. It reveals when and where people undertake activities and how these activities are spatially and temporally structured. An individual’s activity structure is defined as follows:

(2) $$tr_{as}=\left\{\left({\;\mathrm{Actloc}}_{1},t_{1}\right),\ldots \ldots ,\left({\;\mathrm{Actloc}}_{i},t_{i}\right),\ldots \ldots ,\left({\;\mathrm{Actloc}}_{n},t_{n}\right)\right\}$$

where Actloc*_i_* means the individual’s semantic location type at time point *i*, which reflects what activity the individual performs. Semantic location type is represented by the type of point-of-interest (POI). A point of interest (POI) is any point of geographic significance on a map; it can be a hotel, building, bus stop, and so on. Each POI exhibits four basic features: name, function type, address information, and longitude and latitude [30]. Actloc*_i_* is determined by the POI-type where the user is at time point *t_i_*. According to the literature [31], we divide the study area into 100 × 100 one-meter grids, and the main POI-type of each basic geographical unit serves as the semantic location type of the unit. If the location point at time point *t_i_* falls into the unit, then Actloc*_i_* is set as the POI-type of this unit. Referring to the POI classification by AutoNavi Map (AutoNavi Software Co., Ltd., Beijing, China), 14 categories of POI-type are used in this study: industrial park, entertainment, home, parking, bus station, company, hospital, hotel, organization, education, tourist place, shopping, financial and restaurant. Each of the grid cells is assigned one of the 14 POI as its dominant activity type. Two indices (frequency density *F_i_* and ratio *C_i_*) are used to determine the POI-type of a grid unit, which is shown in Equations (3) and (4):

(3) $$F_{i}=\frac{n_{i}}{N_{i}}\left(i=1,2,\ldots ,14\right)$$

(4) $$C_{i}=\frac{F_{i}}{\;\sum_{i=1}^{14}F_{i}}\times 100\%\left(i=1,2,\ldots ,14\right)$$

where *i* represents the POI-type, *n_i_* is the number of type *i* POIs in a grid unit, *N_i_* is the total number of type *i* POIs in the study area, *F_i_* is the frequency density of type *i* POIs in a grid cell, and *C_i_* is the proportion of the frequency density of type *i* POIs in a grid cell. The type of this unit is the same as *i* of the maximum *C_i_* [32]. The classification result of the study area based on this method is shown in Figure 2.

An activity structure entails the information about individual activity variation at different times of a day and *tr_as_* is a sequence of semantic location types over time. The representation of an activity structure needs to encode the meaning of an activity pattern. It needs to be easy to calculate for examining the relationships between activity structure and exposure level at the activity structure group level. The study used one-hot encoding to encode the activity type feature and generate a 14-dimensional feature. One-hot encoding is a representation of categorical variables as binary vectors. Specifically, it refers to splitting the column which contains numerical categorical data into many columns depending on the number of categories present in that column. Each column contains “0” or “1” corresponding to which column it has been placed [33]. It is a form of word expression that maps words from a symbolic form to a vector form, and it can handle discrete numerical features and expand the features. Moreover, mapping discrete features to Euclidean space through one-hot encoding will make the distance calculation between features more reasonable. In the subsequent regression model and clustering algorithms, the calculation of the distance between features is very important. Based on this idea, an individual’s activity structure could be analogized as a sentence, and each activity type could be analogized as a word in the sentence. The one-hot strategy is utilized to represent the meaning of activity structure and *tr_as_* is transformed to a one-hot vector.

For a total of 37 time periods, each time period needs to use 14 digits of 0 or 1 to represent the activity an individual conducts. We define the vector space of a daily activity structure for individual *i* as *S_i_* as:

(5) $$S_{i}=\{\left(a_{1},a_{2},\ldots ,a_{m}\right)\in \left\{0,1{\}}^{m}\in \left[*\right]R^{m},m=518\right\}$$

The dimension of the vector is 518, which is the product of the total time periods and the total number of activity types. The time period is represented as *t* ∈ {1,…,37} and the activity type is represented as *l* ∈ {1,…,14}. For *j* = *t* + 37 × (l − 1), *t* ∈ {1,…,37} and *l* ∈ {1,…,14}, *a_i_* = 0 or 1, depending on if the individual is conducting activity *l* in time period *t*. (*a_1_, a_2_,…a_m_*) satisfies Constraint (6).

(6) $$\overset{14}{\underset{l=1}{\sum }}\left(a_{t}+37*\left(l-1\right)\right)=1$$

Therefore, assuming that the set of activity structure of all users is *S*, then *S* = {*S_1_, S_2_, …, S_n_*} and *n* is the total number of the sample population.

### 3.3. Estimation of Individual’s PM_2__.__5_ Exposure

Since human activities are dynamic in time and space, taking the temporal and spatial factors into account in the calculation of personal exposure value can obtain more accurate results. An individual’s PM_2__.__5_ exposure is usually calculated based on the person’s space-time trajectory. The PM_2__.__5_ value of each location point of the person’s trajectory needs to be obtained. Thus, the interpolated PM_2__.__5_ concentration layers at different times in the study area need to be generated first. Kriging interpolation is a geospatial estimation method, which has been widely used in remote sensing data processing, geology, hydrology and other fields [34]. Since the kriging interpolation algorithm is suitable for regionalized variables with spatial correlation, we perform Kriging based on hourly PM_2__.__5_ collected from the 22 monitoring sites on 23 March 2019, so as to create an interpolated PM_2__.__5_ map in the study area. In general terms, the method of Kriging interpolation is expressed as follows:

(7) $$\hat{Z}\left(s\right)=\overset{n}{\underset{i=1}{\sum }}\lambda_{i}Z\left(s_{i}\right)$$

where $\hat{Z}\left(s\right)$ is the PM_2__.__5_ concentration estimated at an unknown location point s, *n* is the number of all known location points, *Z(s_i_)* is the PM_2__.__5_ observation value at point *s_i_* and$\;\lambda_{i}$ is the weight corresponding to *Z(s_i_)*. At the same time, to meet the two adjustments of unbiasedness and optimality, it is necessary to determine the corresponding weight coefficients through Equation (8) where $\gamma \left(s_{i}-s_{j}\right)$ and $\gamma \left(s_{j}-s\right)$ are the variograms, μ is the Lagrangian coefficient:

(8) $$\left\{\begin{array}{c} \;\sum_{i=1}^{n}\lambda_{i}\gamma \left(s_{i}-s_{j}\right)+\mu =\gamma \left(s_{j}-s\right)\\ \sum_{i=1}^{n}\lambda_{i}=1 \end{array}\right.$$

**Figure 3:**
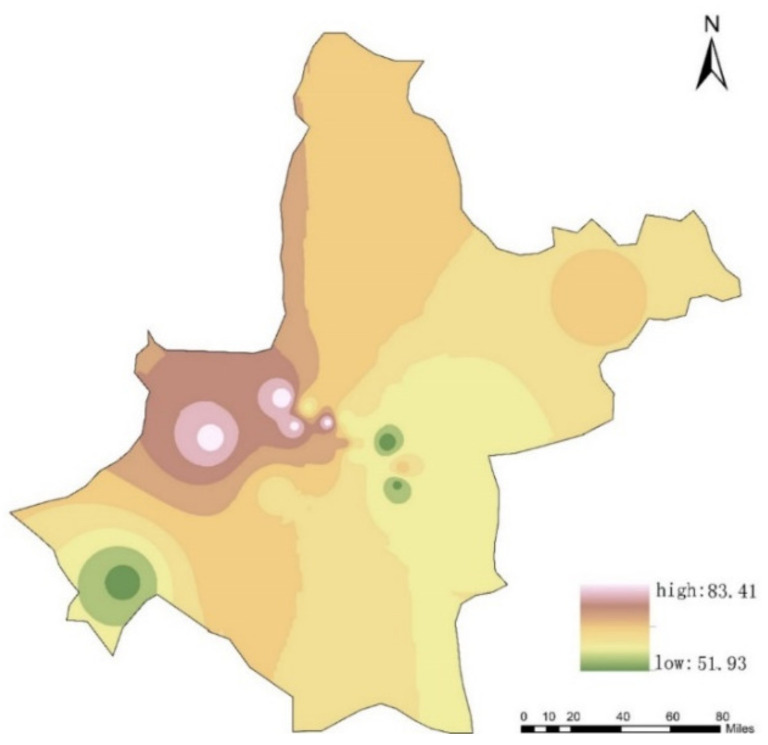
PM_2__.__5_ concentration distribution map of the study area.

Figure 3 shows the PM_2__.__5_ average concentration distribution map of the entire Wuhan city.

We extract each individual’s exposure to PM_2__.__5_ by identifying the intersection between his or her daily movement trajectories and the PM_2__.__5_ concentration layers. Individual exposure to PM_2__.__5_ is obtained as the average of these thirty-seven 30-min interval exposure values, which captures the variations in a person’s PM_2__.__5_ exposure due to changes in his or her location and pollution concentrations in the environment. Personal exposure of the 15,120 residents in Wuhan is estimated using Equation (9).

(9) $$PE=\sum_{t=1}^{37}C_{t}/37$$

where, *C_t_* represents the PM_2__.__5_ concentration of the location at time point *t*, and *PE* represents the personal exposure of an individual in μg/m^3^.

### 3.4. Exploring the Relationships between Individual Activity Structure and Exposure Level

To investigate whether human activity structure and PM_2__.__5_ exposure level are correlated at both the individual and group levels, we design two distance matrices: the activity structure distance matrix and the PM_2__.__5_ exposure distance matrix between individuals. Then, the correlation between the distance of activity structure and the distance of PM_2__.__5_ exposure between two individuals is evaluated. The assumption is the shorter the distance of the activity structures between two individuals, the closer are the PM_2__.__5_ exposure levels between them, and vice versa. Further, the sample individuals can be clustered into several groups with similar activity structures. Then, based on this assumption, we further hypothesize that people with similar activity structures (shorter activity structure distance) are more likely to form an activity structure cluster and experience similar exposure to PM_2__.__5_ pollution, while people having greatly different activity structures are more likely to fall into different activity structure clusters and have considerable differences in their PM_2__.__5_ exposure at the group level. If the assumption is supported, human activity structure does impact people’s PM_2__.__5_ exposure level to a greater extent and further examinations on how different activity structures influence people’s exposure should be conducted. 

**Figure 4:**
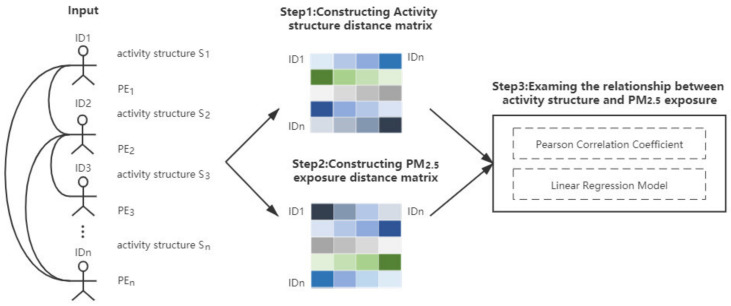
The workflow of measuring the relationship between activity structure and PM_2__.__5_ exposure.

Figure 4 shows the detailed workflow of measuring the relationship between activity structure and PM_2__.__5_ exposure. Based on the method described in Section 3.2 and Section 3.3, the vector representation of individual *i’s* activity structure *S_i_* and the PM_2__.__5_ exposure *PE_i_* could be prepared for each individual as the input.

First, the activity structure distance matrix *M_a_* is constructed by calculating the activity structure distance between any pair of individuals. The dimension of *M_a_* is *m* (15,120), which is equal to the number of the sample population. Thus the size of *M_a_* is *m × m*. The distance between the activity structures of two individuals is measured by cosine similarity. Due to the long and sparse features of the activity structure vector, cosine similarity distinguishes the difference in the vector direction and is often used to measure document similarity in text analysis, which could fully consider the temporal dimension feature of human activity structure. The similarity degree is measured by the cosine of the angle between two vectors which determines whether two vectors are pointing in roughly the same direction. Hence, cosine similarity is used to calculate the degree of similarity in the activity sequences between two individuals. The closer the cosine value is to 1, the closer the angle is to 0 degrees, indicating more similarity in the activity structures of the two individuals. The distance between the activity structures of two individuals is expressed in Equation (10) as follows:

(10) $$D_{ij}=1-\frac{\left(S_{i}\;\cdot \;S_{j}\right)}{∥S_{i}∥∥S_{j}∥}$$

where *S_i_* represents the activity structure vector of individual *i* and *S_j_* represents the activity structure vector of individual *j*. The expression after the minus sign indicates the cosine similarity between the *S_i_* and *S_j_*. *AD_ij_* represents the activity structure distance between individual *i* and individual *j*. 

Then, the personal exposure distance matrix *M_e_* is constructed by calculating the exposure distance between any pair of individuals. The dimension and size of *M_e_* are the same as *M_e_*. The distance between the personal exposure of two individuals is expressed as *ED_ij_* in μg/m^3^ in Equation (11). *PE_i_* represents the personal exposure value of individual *i* and *PE_j_* represents the personal exposure value of individual *j*:

(11) $$ED_{ij}=\left|PE_{i}-PE_{j}\right|$$

Finally, the quantitative analysis is conducted between *M_a_* and *M_e_* and (*AD_ij_, ED_ij_*) is the basic unit consisted of the corresponding elements from *M_a_* and *M_e_*. Using these two matrices, we employ the Pearson correlation analysis to measure the strength of the association between the two variables of activity structure distance and PM_2__.__5_ exposure distance and linear regression to examine whether the statistical relationship between them is linear.

## 4. Results

To explore the relationship between human activity structure and PM_2__.__5_ exposure, in this section we cluster the individuals in our sample into different activity structure groups and discuss the relationships between the two at the inter- and intra-group levels.

### 4.1. Identification of Groups with Different Activity Structures

**Figure 5:**
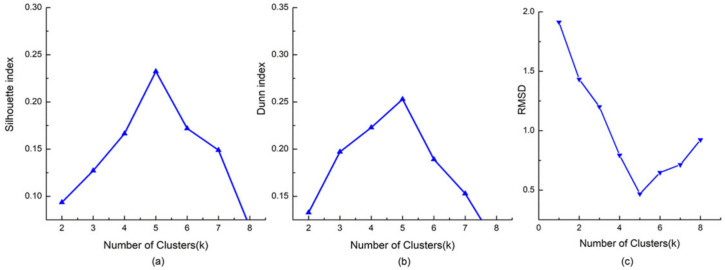
(**a**) the Dunn index (**b**) the Silhouette index and (**c**) the RMSD for different number of clusters.

In this subsection, we generate the clusters of similar individual activity structures by applying the K-means algorithm [35] (the algorithm is provided in Appendix A). One problem that needs to be taken into account in the clustering process is how to determine the most suitable number of clusters. The Dunn index [36] and the Silhouette index [37] are utilized in this paper to evaluate the clustering results [38]. The Dunn index mainly reflects the compactness and separation of clusters, and the silhouette index reflects the rationality of clustering. The higher the values of the two indices, the better the clustering result. Figure 5 shows the changing trend of the Dunn index and the Silhouette index when choosing different cluster numbers. Note that both indices indicate that a relatively stable clustering effect can be achieved when the number of clusters is five. In addition, RMSD [39] which is defined as the sum of the root mean square deviations of cluster elements from the corresponding cluster center over clusters are utilized to characterize the homogeneity within clusters. It can be seen that the RMSD value decreases as the number of clusters increases and reaches the lowest value when the number of clusters is five and then increases again. It also shows that five clusters are suitable and produce the largest improvement in cluster performance. Therefore, the sample is clustered into five groups.

The activity structure is organized through a set of hierarchically ordered places that have a particular meaning for an individual [40]. Excluding home, the major place is work or school or a place where a major regular activity occurs. The places where people spend their leisure time and socialize with others are called the secondary places (i.e., shops, cafes, bars, restaurants, parks, etc.) [41]. In this paper, we mainly identify the major place and the secondary place to compose and analyze the activity structure. For the convenience of discussion, the main characteristic of the activity structure of each group is portrayed. The major place is utilized to identify the social character for each cluster.

Therefore, for each activity structure group, the ratio of the visited number of each POI-type to the total number of all visited POI-types at each time period is calculated and the POI-type with a percentage larger than 70% is defined as the major type that the major activity takes place. The corresponding relationship between the social character of each activity structure group and POI category is defined in Table 1.

The POI-types of “industrial park”, “home”, “education”, “company” and “tourist place” listed in Table 1 for groups 3–5 are consistent with the POI category used by AutoNavi Map (AutoNavi Software Co., Ltd., Beijing, China). Considering that “company” defined by AutoNavi Map is relatively broad, this paper defines “industrial park” as the enterprises that have a serious effect on the surrounding environment, such as metallurgy and chemical industries, minerals, and construction, while other companies such as network technology, advertising and decoration, commercial trade, high-tech enterprises, and so on are defined as “company”. In this way, it is possible to distinguish between factory workers and office workers.

Five distinctive activity structure clusters are identified and defined as “factory-centered activity structure”, “office-centered activity structure”, “home-centered activity structure”, “outgoing-centered activity structure” and “education-centered activity structure” which represents the group of “factory workers”, “office workers”, “stay-at-home”, “adventurers” and “education-related” respectively. People in the group with a much higher proportion of factories than that of other POIs spend most of their time at a factory, and thus the group is defined as a “factory-centered activity structure”. Similarly, the groups with much higher proportions of enterprises, home, and education (i.e., schools, museums, art galleries and exhibition) are defined as “office-centered activity structure”, “home-centered activity structure” and “education-centered activity structure”, respectively. Lastly, the group of “outgoing-centered activity structure” is defined when the total proportion of “shopping”, “leisure entertainment”, and “green space and parks” is dominant. For the simplicity of expression, “factory workers”, “office workers”, “stay-at-home”, “adventurers” and “education-related” will be used in the later sections to represent each activity structure group.

It is noteworthy that the average time proportion of staying at home for the “stay-at-home” group reaches 90%, which means this group of people mainly stay at home and rarely perform out-of-home activities. Since the activities of staying at home account for a very high percentage and thus the residential location (instead of the activity structure) may play a decisive role in the individual exposure level of people in this group. Therefore, the “stay-at-home” group will be excluded in later discussion. In other words, only four activity structure groups—“factory workers”, “office workers”, “adventurers” and “education-related” will be taken as the whole sample and analyzed.

### 4.2. Correlation Analysis of Activity Structure and PM_2__.__5_ Exposure Level

The Pearson correlation coefficient between the distance of activity structure and the distance of PM_2__.__5_ exposure is 0.78 (*p* < 0.01). With a *p*-value < 0.01, the correlation coefficient is statistically significant. The results indicate that there is a strong correlation between individual activity structure and individual PM_2__.__5_ exposure.

**Figure 6:**
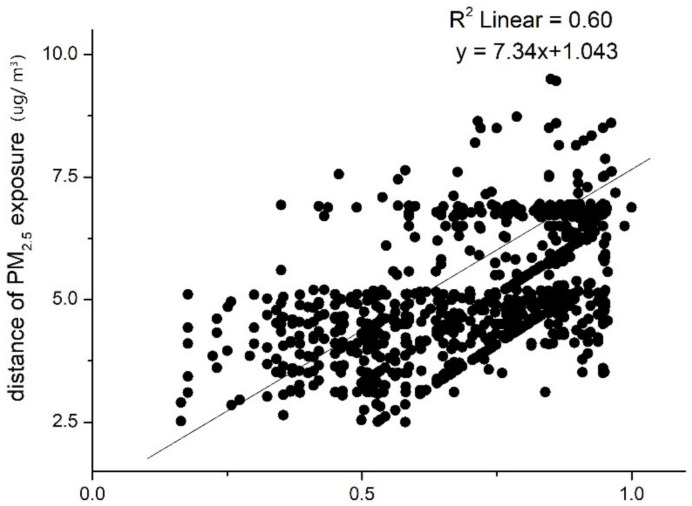
Relationship between PM_2__.__5_ exposure and activity structure.

**Figure 7:**
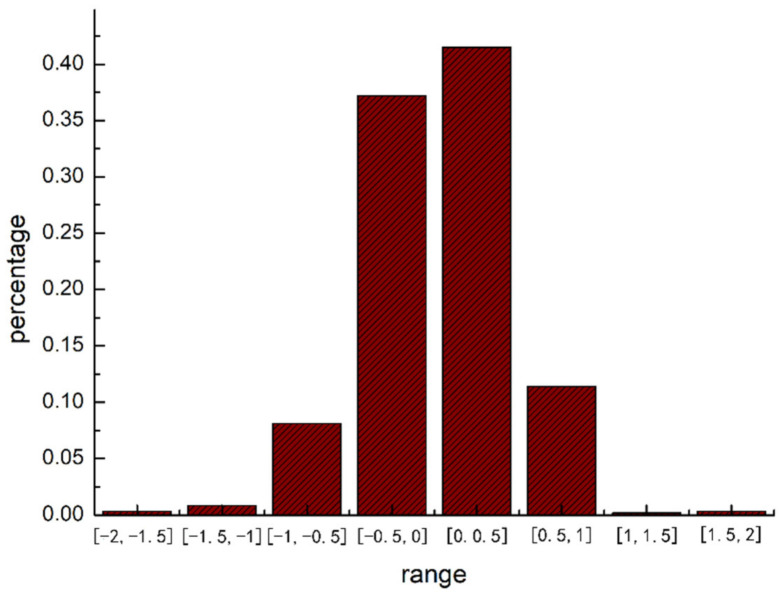
Distribution of residuals.

Then, a linear regression model is estimated and the results show a positive relationship between the distance of PM_2__.__5_ exposure and distance of activity structure vector (with a slope of 7.34), which is shown in Figure 6. The residual distribution is illustrated in Figure 7, which indicates that 78.7% of the absolute residuals are less than 0.5, and 98.2% of the absolute residual is less than 1. This indicates that in most cases, the estimated distance of PM_2__.__5_ exposure value is close to the actual distance of PM_2__.__5_ exposure value.

The above results also indicate that the shorter the distance of activity structure between two individuals, the shorter is the distance of PM_2__.__5_ exposure between them. On the contrary, the longer the distance of activity structure between two individuals, the longer the distance of PM_2__.__5_ exposure between them. Based on the properties of clustering, people with similar activity structures are more likely to form a similar activity pattern and may have similar exposure to PM_2__.__5_ pollution, and vice versa. From this perspective, the structure of human activities influences the level of individual exposure to a certain extent.

We further examined the Pearson’s correlation coefficient for each activity structure group, the results are shown in Table 2. All the value of the Pearson’s *r* for each group is larger than 0.70. The value of Pearson’s *r* for the factory workers and the education-related group is 0.85 and 0.81, which shows that for the two activity structure groups, there is a very strong correlation between activity structure and exposure level. For the adventurers and office workers, the correlation between activity structure and exposure level is a little lower compared with the former two groups, with the value of *r* still higher than 0.73. In Table 3, the value of R^2^ on the entire sample is 0.60, which means the activity structure distance could interpret 60% of the variance in the exposure level distance. Similarly, the values of R^2^ for the factory workers and education-related group are higher than the office workers and adventurers group.

The above results also cogently answer the question we posed at the beginning of this paper: whether individuals’ activity structure influences their PM_2__.__5_ exposure? The connection between people’s activity structure and their PM_2__.__5_ exposure has been established quantitatively hereto, which provides the basis to further analyze how different activity structures impact people’s exposure levels at the inter- and intra-group levels.

### 4.3. Inter-Group Relationships between Activity Structure and Exposure Effects 

This subsection examines whether groups with different activity structures have different exposure levels and whether the relationship between the distance of activity structure and the distance of PM_2__.__5_ exposure also applies to these groups. 

**Figure 8:**
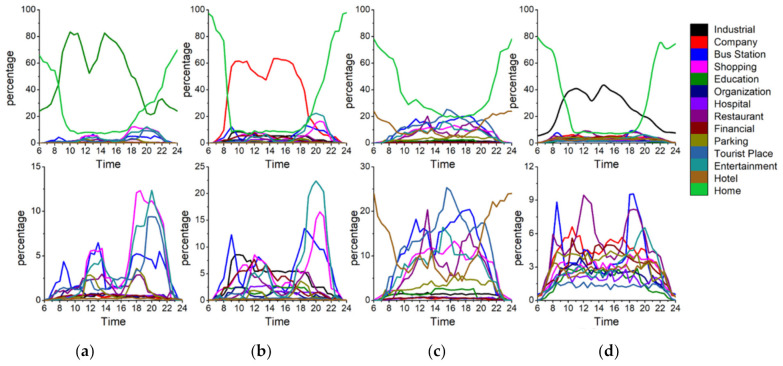
Activity structure of the four groups. (**a**) Factory workers (**b**) Office workers (**c**) Adventurers (**d**) Education-related.

**Figure 9:**
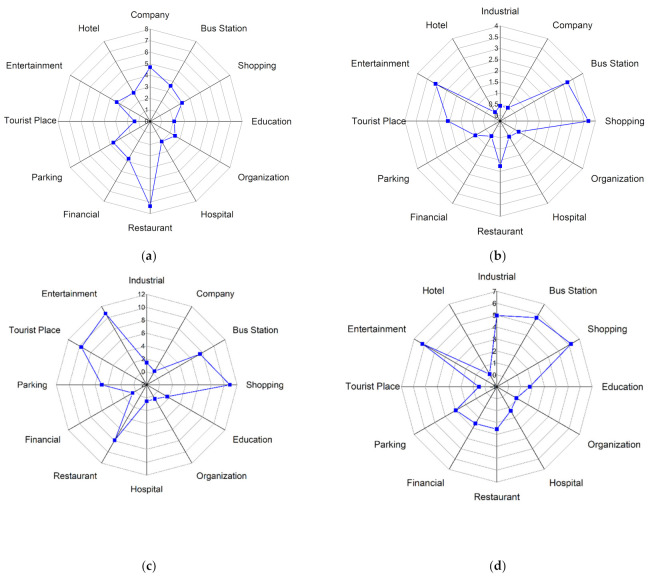
Secondary activity structure of the four groups using radar graph. (**a**) Factory workers (**b**) Office workers (**c**) Adventurers (**d**) Education-related.

First, Figure 8 shows the activity structure of the four distinctive groups. The horizontal axis represents time (hour) and the vertical axis represents the percentage of the number of users appearing at a specific POI-type among the total number of users. Each line represents the percentage of a specified POI-type. Thus the top line represents the main activity and the other lines represent the secondary activities of a specific group. Note that the four groups have distinctive activity patterns. Figure 9 represents a radar graph that shows the activity structures of the secondary activities of the four groups.

Factory workers refer to people who spend most of their time at a factory. The proportion of factory as the primary activity places starts to rise around 6:00 a.m. which is the earliest among the four groups and declines at 4:00 p.m. They undertake secondary activities actively at noon and evening, but less than those of office workers and adventurers. Figure 9a indicates that the main secondary activity places of factory workers consist of restaurants, shopping and entertainment-related places. Among these activity places, the restaurant has the highest proportion (9%). It means that the structure of factory workers’ activities is relatively simple and monotonous. In addition to working in a factory, eating out is the most important activity for this group. It is also noteworthy that this group rarely visits green spaces and parks (1%) or education-related places (2%) which would reduce their PM_2__.__5_ exposure and benefit their health.

Office workers mainly refer to the people whose workplaces are the enterprises such as high-tech companies, financial institutions, advertising and decoration, commercial trade, and so on. Figure 8b shows the activity structure of this group. Most of them leave home during 7:00–8:00 a.m. and return home from work at 5:00–8:00 p.m. Their secondary activities after work are vibrant and plentiful. Office workers are usually white-collar workers with a mid-to-high income level, and hence they are more willing to go out for social, recreational and leisure activities after work. The main secondary activity places of this group consist of entertainment avenues, shopping centers, restaurants, bus stations, parking places and industrial parks. The proportion of shopping and leisure activities for office workers is surpassed only by that of the adventurer group and is much higher than factory workers and education-related workers. These two kinds of activities are characterized by two obvious peaks. The first peak appears around 12:00 a.m., which is associated with the noon break. The second peak appears around 8:00 p.m. The proportion of dining-out activities is a little lower when compared with entertainment activities. The first peak appears near 1:00 pm and the second peak appears around 5:00 p.m. It is noteworthy that this group rarely visits education-related places (less than 2%) and green spaces and parks (less than 1%), which means that the use of green spaces is very low in this group.

Adventurers mainly refer to people who visit public places for relaxation and recreation (i.e., tourist places, green spaces, parks, and so on) as their main activities. Figure 8c shows the activity structure of this group which mainly includes tourists and local residents who like to undertake outing activities. According to the places where they leave in the morning, about 65% of this group belong to the local residents and approximately 15% of this group belong to the tourists. The home ratio (proportion of staying at home) in the morning reaches a trough around 11:00 a.m., which shows that compared with ordinary workers, the travel time of people who like to go out is relatively flexible. They usually avoid the morning rush hour and undertake outing activities when traffic is smooth. The home ratio in the evening usually starts to rise at 6:00 p.m, which is one hour later than that of the ordinary workers. And the upward trend is relatively gentle, especially between 6:00–8:00 pm, which means that the outing activities of adventurers usually last until the late evening. The main activity structure of adventurers consists of green spaces and parks (15%), entertainment avenues (9%), bus stations (10%), shopping centers (10%) and restaurants (8%). This group rarely visits industrial parks and companies.

The education-related group mainly includes education practitioners and students. They start education-related work during 7:00–8:00 a.m. and finish working around 6:00 pm. There is a small peak around 8:00–10:00 pm, which means some people still have to do education-related work at night (i.e., teaching or attending evening classes). The peak value of the proportion of education and cultural places for this group is close to 80%, which is the highest for the main activity among the four groups. It also means that members of this group perform the fewest secondary activities. The time secondary activities occur is at noon and in the evening. Figure 9d represents the activity structure of the secondary activities of this group. The main places this group visit are entertainment avenues (3%), greens and parks (3%), restaurants (2%), shopping-related places (5%) and bus stations (3.5%). This group rarely visit industrial parks, companies and hotels.

**Figure 10:**
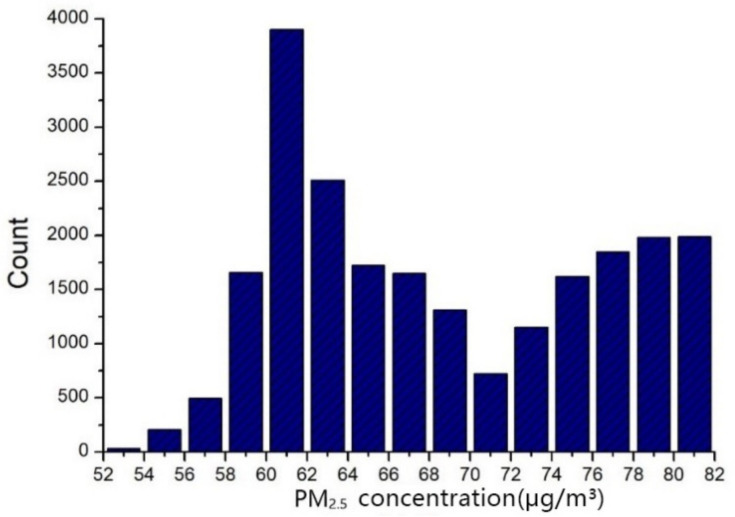
The daily personal PM_2__.__5_ exposure level of 15,120 residents in Wuhan.

**Figure 11:**
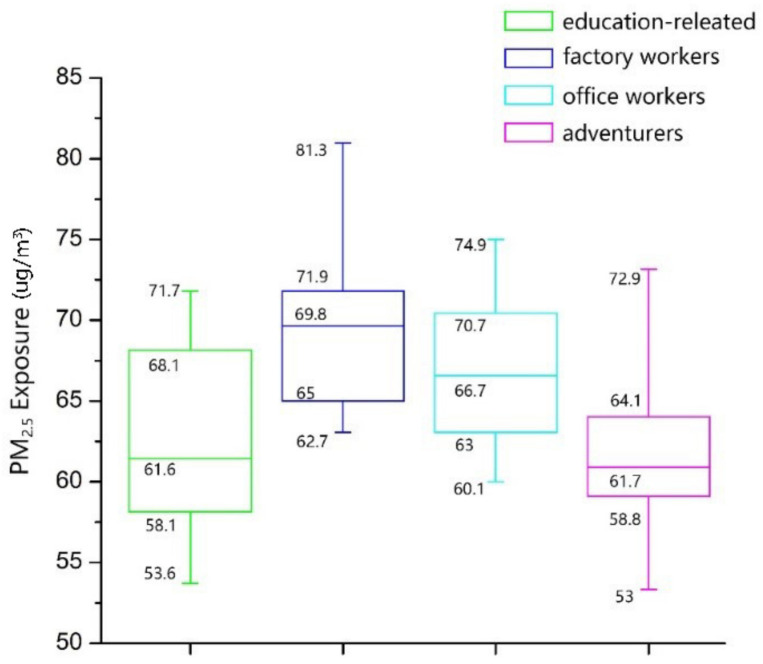
The box plot represents three quartiles of PM_2__.__5_ exposure of the four activity structure groups.

The above results indicate that the characteristics of the activity structure for each group are distinctive. Second, we examine whether the PM_2__.__5_ exposure levels of the four activity structure groups are different. Individual exposure level was calculated according to the average PM_2__.__5_ concentration at each time interval. The mean personal PM_2__.__5_ exposure level of the sample residents is 67.67 μg/m^3^ (range: min 51.93–max 83.41). The personal exposure levels of the 15,120 residents are shown in Figure 10. The box plot in Figure 11 represents three quartiles of PM_2__.__5_ exposure of the four activity structure groups. PM_2__.__5_ exposures of the four activity structure groups show a clear sequence from high values to low. They are factory workers, office workers, education-related, and adventurers. Specifically, the minimum value, maximum value and mean value of PM_2__.__5_ exposure for the factory workers are 62.7, 81.3 and 69.0 μg/m*^3^*. All these three values are the highest among the four groups. It indicates that, on the whole, factory workers tend to be exposed to the highest level of PM_2__.__5_ pollution among the four activity structure groups and the overall exposure risk of this group is the highest among them. This is in line with our expectations. According to the activity structure of this group, factory workers have long working hours and suffer from the high pollution of their work environments. They often have difficulty in accessing high-end facilities and rarely have the opportunity to reduce their daily exposure level [9].

Next to the factory workers, the minimum value, maximum value and mean value of PM_2__.__5_ exposure for office workers are 60.1, 74.9 and 66.2 μg/m^3^. Thus, it indicates that the group of office workers as a whole still tends to have a relatively high exposure risk due to their high mobility and diversified activities. Then, the minimum value, maximum value and mean value of PM_2__.__5_ exposure for the education-related group are 53.6, 71.7 and 61.7 μg/m^3^. This group tends to have relatively low exposure risks. This is mainly because the workplaces of this group are educational and cultural places, where the vegetation coverage rate and the overall greening level are relatively high. Lastly, the minimum value, maximum value and mean value of PM_2__.__5_ exposure for adventurers are 53.0, 72.9 and 60.7 μg/m^3^. On the whole, this group tends to have the lowest exposure risk because of its highest levels of green space usage among the four groups. In general, the PM_2__.__5_ exposures of factory workers and office workers are higher, while the PM_2__.__5_ exposures of the education-related group and adventurers are lower. 

To further verify whether the mean PM_2__.__5_ exposure levels of the four activity structure groups differ significantly from each other, an ANOVA test [42] which compares the means of a continuous variable in two or more independent comparison groups was performed. The results are shown in Table 4. It can be seen that the between-group and within-group mean square deviations are 45,614 and 9.20 respectively. The F-value (4596.97) is much larger than the critical value of F (2.6056) when the level of significance is 0.05. This huge F-value is strong evidence that the null hypothesis (the four activity structure groups having equal mean PM_2__.__5_ levels) should be rejected. Meanwhile, *p* = 0.004 (<0.05) means the result is statistically significant and the mean PM_2__.__5_ exposure levels of the four activity structure groups differ significantly from each other.

Then, the post-hoc procedure of the Scheffé test [43] was conducted to further examine which group pairs’ PM_2__.__5_ exposure differ significantly from each other. The result is shown in Table 5, which indicates that the F-value between the adventures and education-related groups is less than F-Critical (7.8168), and the F-value between any other pairs of activity structure groups is all greater than F-Critical. This indicates that no significant difference exists between the mean PM_2__.__5_ of adventures and education-related groups, while a significant difference exists between the mean PM_2__.__5_ of the rest of the activity structure group pairs.

These results indicate that different activity structure groups do experience different levels of PM_2__.__5_ exposure. In other words, at the inter-group level, different activity structures affect people’s exposure level and daily activity structure does have a certain influence on people’s exposure.

**Figure 12:**
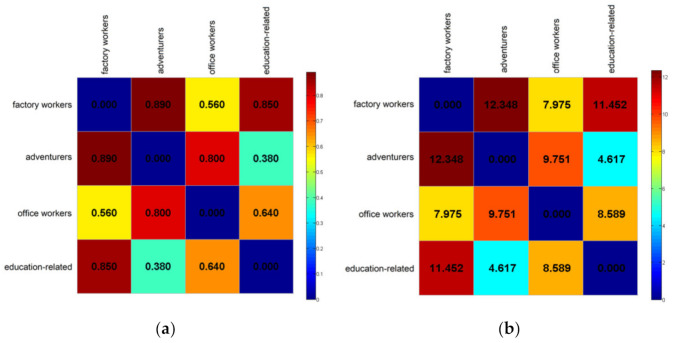
Activity structure distance matrix (**a**) and PM_2__.__5_ exposure distance matrix (**b**) among the four activity structure groups.

Finally, we examine the activity structure distance and PM_2__.__5_ exposure distance among the four groups. Figure 12 represents the activity structure distance matrix and PM_2__.__5_ exposure distance matrix among the four activity structure groups. The activity structure distance between the two groups is calculated using the average distance between them, and the PM_2__.__5_ exposure distance uses the F-value of the Scheffé test. As shown in Figure 12a, for factory workers, the distance of activity structure between this group and office workers is the shortest, followed by the education-related group and finally the adventurer group. The distance of PM_2__.__5_ exposure between factory workers and the other three groups also follows this order. Specifically, the activity structure for factory workers and office workers are the most similar (0.83), and the distance of PM_2__.__5_ exposure between these two groups is the shortest (7.97). On the contrary, the activity structure for the factory workers and the adventures are the most dissimilar (0.89). Therefore, the distance of PM_2__.__5_ exposure between these two groups is the longest (12.34). For adventures, this group is most similar to the education-related group, followed by office workers and finally factory workers in terms of activity structure. The distance of PM_2__.__5_ exposure among them also follows the same order. In general, the distance sequence of PM_2__.__5_ exposure corresponds with that of the activity structure among the four activity structure groups. This indicates that the relationship between the activity structure and PM_2__.__5_ exposure is also true at the level of activity structure group. That is to say, the smaller the distance of activity structure between two activity structure groups, the closer is the distance of PM_2__.__5_ exposure between them. On the contrary, the longer the distance of activity structure between two activity structure groups, the longer is the distance of PM_2__.__5_ exposure between them.

### 4.4. Intra-Group Relationship between Activity Structure and Exposure Effects

This subsection further observes the interaction relationship between activity structure and exposure level inside each activity structure group in the reverse direction. We first divide the PM_2__.__5_ exposure level into three categories: high (ranges from 70.20 to 81.3 μg/m^3^), medium (63.54 to 70.19 μg/m^3^) and low (53 to 63.54 μg/m^3^) by clustering the PM_2__.__5_ exposure value. Then, the activity structures at different exposure levels for each group are examined whether people at different exposure levels have different detailed characteristics within each group.

#### 4.4.1. Factory Workers

**Figure 13:**
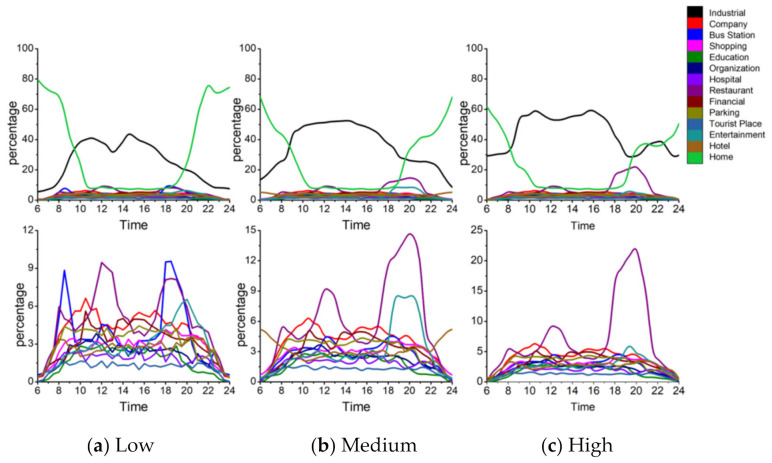
Factory workers’ daily activity structures at different exposure levels.

**Figure 14:**
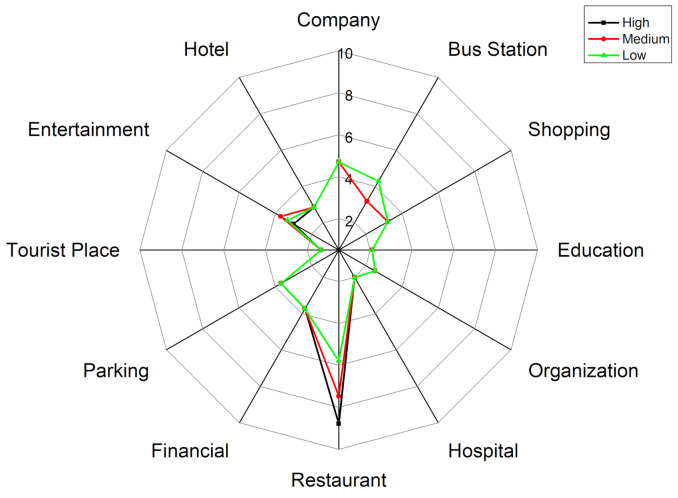
The activity structures of the secondary activities of factory workers at different exposure levels.

Figure 13a–c show the activity structure at high-, medium- and low exposure levels. The three charts in the upper row describe the overall activity structure of the group and the three charts in the lower row depict the details of the curves that represent the secondary activities in the first row. Figure 14 is a radar graph that shows the activity structure of the secondary activities of factory workers at different exposure levels.

There are differences in the working hours at different exposure levels. The proportion of factory starts to rise around 6:00 a.m. At high exposure levels, the factory curve falls to 28% at the bottom during 6:00–8:00 p.m. After 8:00 p.m., the curve shows an upward trend and rises to 40%. It can be explained that 40% of the factory workers still have to return to the factory to work. They may be overtime workers or night-time workers. At medium exposure level, after 4:00 p.m., the curve shows a downward trend and there is no trend of recovery, reaching a trough (25%) at 10:00 p.m. At low exposure level, it also shows a rising trend starting from 4:00 p.m., and the downward trend continues until 10:00 p.m. The results indicate that the working hours of this group at low- and medium exposure levels are significantly shorter than those at high exposure levels. In other words, by reducing the working hours or the time spent in factory, the individual exposure of factory workers is reduced to a great extent.

The proportion of home also differs at different exposure levels. At high exposure levels, the proportion of home shows an upward trend after 7:00 p.m., reaching a peak (37%) at 9:00 p.m. and then slightly decreases. At medium exposure level, it shows a sharp increase since 6:00 p.m., reaching 43% at 10:00 p.m., and there is no trend of falling back. At low exposure level, the curve increases sharply since 6:00 p.m., reaching 78% at 10:00 pm. This indicates that the proportion of returning home after work increases significantly with the decrease of exposure level and the choice of going home earlier helps reduce the personal exposure level of factory workers to a great extent.

There are also differences in recreational activities for factory workers at different exposure levels. At high exposure levels, there are no obvious recreational activities except for dining activity which shows two obvious peaks during lunch and dinner time. The shopping and entertainment activities account for a very low percentage, which means the recreational activities are monotonous. This may be due to the limitation of working hours and they have very little time to take non-working activities out. At low- and medium exposure levels, on the one hand, the proportion of factory decreases. The peak proportion of factory at high-, medium- and low-exposure level during 9:00 a.m.–5:00 p.m. are 60%, 51% and 41%. On the other hand, the recreational activities of the factory workers at low- and medium exposure levels start showing a diversified trend. The non-working activities are more abundant than those at high exposure levels. 

Among the factory workers, the activity structures at different exposure levels have differentiation in detail. The results show that the higher the exposure level, the longer the working hours and the more monotonous the types of recreational activities; while the lower the exposure level, the shorter the working hours and the more diverse the types of recreational activities. 

#### 4.4.2. Office Workers

**Figure 15:**
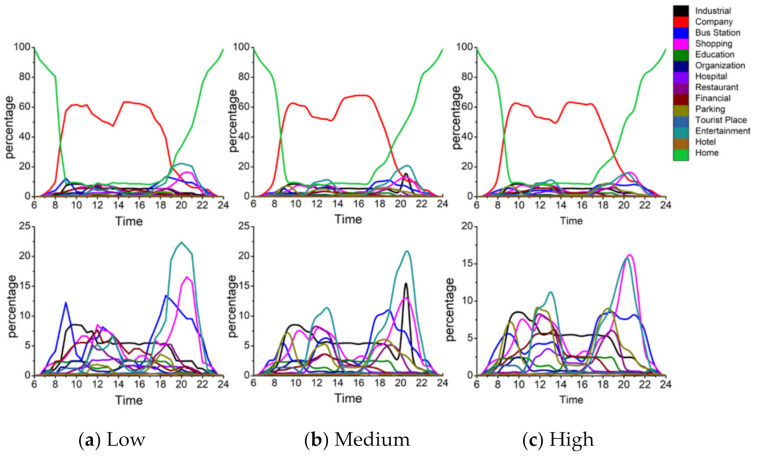
Office workers’ daily activity structures at different exposure levels.

**Figure 16:**
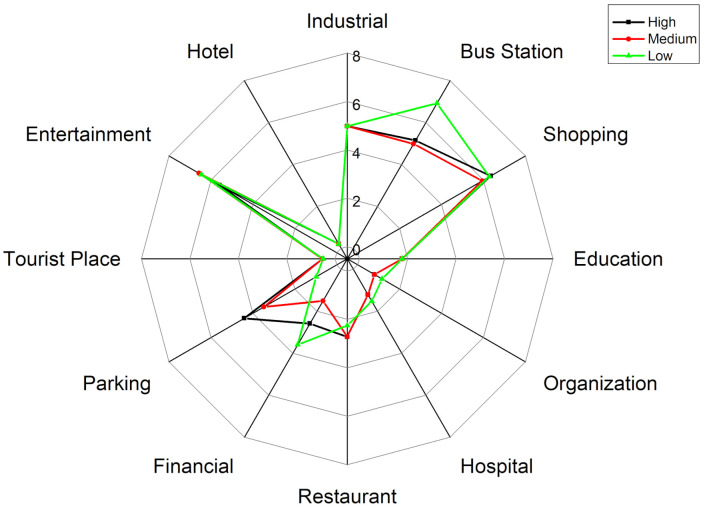
The activity structures of the secondary activities of office workers at different exposure levels.

Figure 15 shows the secondary activity structure of office workers. Figure 16 shows the activity structure of the secondary activities of office workers at different exposure levels.The activity structures of this group at different exposure levels are similar on the whole. Differences in the choice of commuting modes for this group at the three exposure levels are observed. At high exposure levels, the parking curve shows three peaks in the time periods of 7:00–10:00 a.m., 12:00–2:00 p.m., and 5:00–8:00 p.m. This is consistent with the commuting hours in the morning peak, the noon break and the evening peak. The proportion of parking at high exposure level is the highest with three peaks (8%, 10% and 9%). The three peak values are 7%, 5% and 5% at medium exposure level, and are 1%, 3% and 4% at low exposure level. It indicates that with the decrease in the proportion of the use of parking, the exposure level also shows a downward trend. Interestingly, the use of bus stops also shows three obvious peaks in the three corresponding time periods. The three peak values are 6%, 4% and 8% at high exposure level, are 6%, 7% and 12% at medium exposure level, and are 13%, 8% and 14% at low exposure level. It indicates that people with lower exposure levels tend to use bus stops higher, while people with higher exposure levels tend to use parking lots more. In other words, the travel mode of office workers with high exposure levels is mostly private cars while the office workers with low exposure levels often choose public transportation as their commuting mode. In addition, the shopping and leisure activities for office workers are colorful, but the obvious differences in recreational activities among this group at different levels of exposure are not observed.

#### 4.4.3. Adventurers

**Figure 17:**
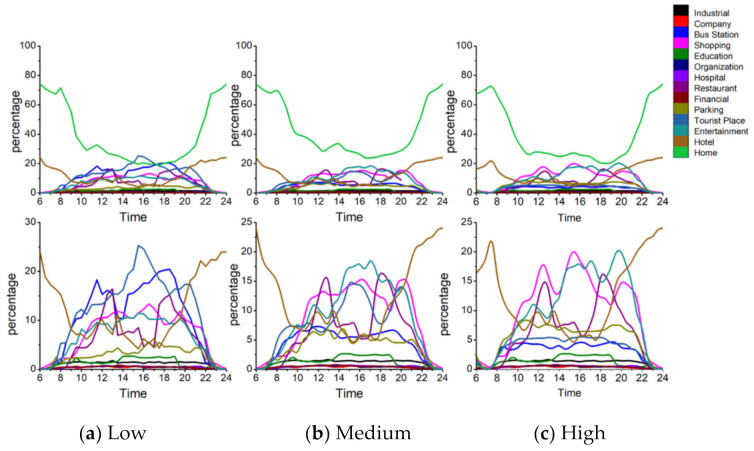
Adventurers’ daily activity structures at different exposure levels.

**Figure 18:**
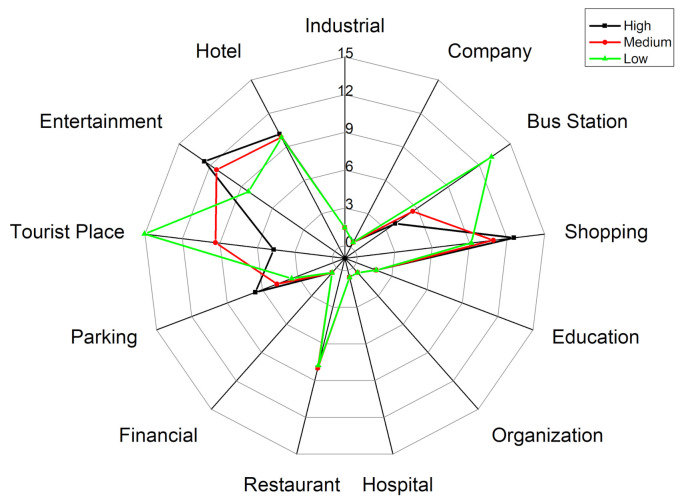
The activity structures of the secondary activities of adventurers at different exposure levels.

Figure 17 shows the structure of the secondary activities of adventurers at different exposure levels. Figure 18 shows the activity structure of the secondary activities of adventures at different exposure levels. From the activity structure point of view, there are obvious differences at the three different exposure levels. For shopping activities, the peak value of the percentage is 20% at high exposure level, 15% at medium exposure level, and 12% at low exposure level. The higher the exposure level, the higher the proportion of shopping activities and vice versa. From the perspective of time, shopping activities fluctuate less at different times during the day and are evenly distributed, which means adventurers would take shopping activities at any time of the day.

The trends of recreational activities during 8:00 a.m.–6:00 p.m. at different exposure levels are similar, but there are differences during 7:00 p.m.–9:00 p.m. at night (the time period with the worst air quality). At high exposure level, the leisure and recreational activities account for the highest proportion, with a peak of 21%; at medium exposure level, the ratio declines and peaks at 13%; at low exposure level, the proportion falls sharply at 10%. Similar to shopping activities, it can be observed that the higher the exposure level, the higher the proportion of leisure and entertainment activities and vice versa.

At different exposure levels, the travel modes are also different. At low exposure levels, the use of public transportation accounts for the highest proportion, reaching two peaks 18% and 20% at 11:00 a.m. and 6:00 p.m.; while the use of public transportation at high exposure levels is very low with an average value of 3%. At the same time, it is also observed that the proportions of parking at high-, medium- and low exposure levels are different (the average proportions are 8%, 5%, and 2% respectively). In other words, at high exposure levels, adventurers tend to take outing activities and travel by car; while at low exposure levels, adventurers mainly use public transportation to travel.

The visiting behaviors to tourist places (i.e., greens and parks) differ at different exposure levels. At low exposure level, the proportion of such activities is at a relatively high level, gradually rising from 8:00 a.m., reaching the first peak (25%) and the second peak (18%) at 3:00 p.m. and 8:00 p.m. and then falling sharply. At the medium exposure level, the trend is similar to that at low exposure levels, but the proportion decreases and reaches the first peak (14%) and the second peak (13%) at 3:00 p.m. and 8:00 p.m. However, at high exposure levels, the ratio is very low, with an average value of 5%. It shows that different ways of using the greens and parks may lead to different exposure results. The adventurers at low exposure level visit more green spaces, while the adventurers at high exposure level rarely visit such places.

Generally, the detailed activity structure of adventurers is different at different exposure levels. Adventurers with high exposure level visit leisure and entertainment, shopping-related places and restaurants with a very high percentage and the main travel mode is the private car. It is noteworthy that the use of green spaces is very low. However, adventurers with low exposure levels are more inclined to visit tourist places and travel by public transportation, but the entertainment and shopping activities are relatively low. 

#### 4.4.4. Education-Related Group 

**Figure 19:**
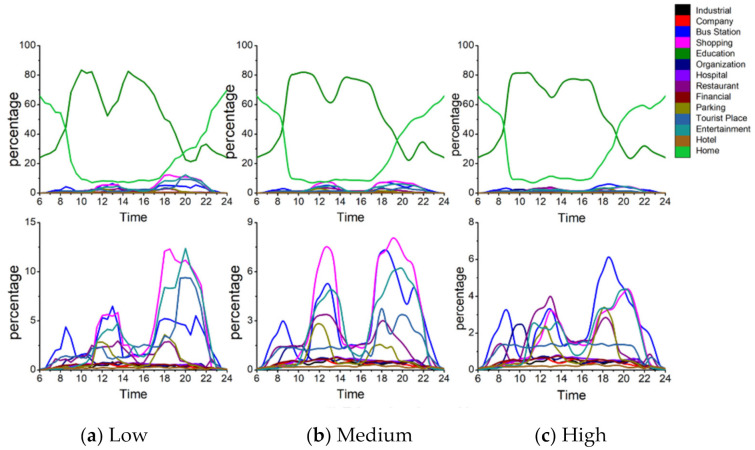
Daily activity structures of the education-related group at different exposure levels.

**Figure 20:**
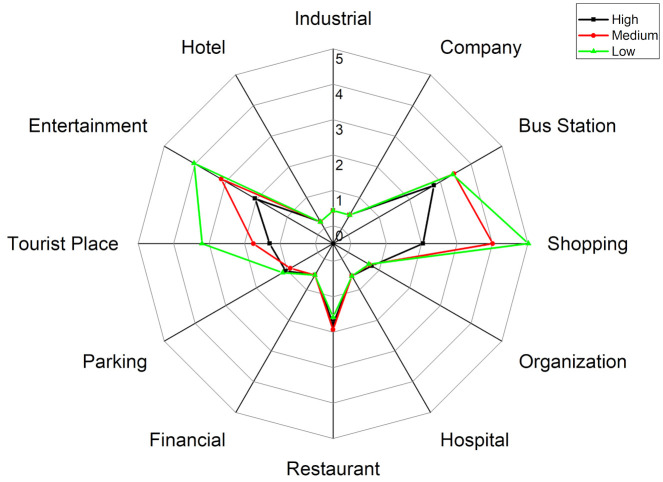
The activity structures of secondary activities of the education-related group at different exposure levels.

Figure 19 represents the structure of the secondary activities of the education-related group at different exposure levels. At high exposure levels, people leave home during 7:00–8:00 a.m., return home during 5:00–6:00 p.m. and do less outing activities. At medium exposure level, people start choosing to go out for leisure and entertainment or shopping in the evening. At low exposure levels, the outing activities are more abundant. For example, the proportion of bus stops and green spaces increases, and the overall proportion of people at home is also far lower than the high- and medium exposure levels. It can be seen from Figure 20, at low exposure level, the education-related group shows stronger vitality and has a higher willingness to go out than those at medium-to-high exposure level, and the places visited appear the characteristic of diversification. However, at high exposure levels, this group shows the opposite trait.

Specifically, as the exposure level decreases, the frequency of outing activities (shopping, entertainment and leisure, bus stops, etc.) gradually increases during the periods 12:00 a.m.–2:00 p.m. and after 5:00 p.m. For example, the peak proportion of shopping activities at the high-, medium- and low levels are 2.9%, 5.8%, and 6.9% at noon, and 3.7%, 6.7%, and 9.7% in the evening hours. The peak proportion of bus stations at the high-, medium- and low exposure levels are 2.7%, 3.7%, and 4.7% at noon, and 5.2%, 5.6%, and 5.6% in the evening hours. The peak proportion of leisure and entertainment at high-, medium- and low exposure levels are 2.8%, 4.3%, 4.8% at noon and 2.4%, 5.4%, and 9.4% in the evening hours. The peak ratio of visiting green spaces at night is 1.5%, 3.2%, and 8.5% at high-, medium- and low exposure levels respectively. Therefore, these results indicate that the characteristics of detailed activity structure differ at different exposure levels. People with high exposure have the lowest proportion of secondary activities and vice versa.

In general, by observing the detailed characteristics of people’s activity structures under different exposure levels in each group, there is indeed a distinction between the detailed activity structures. These results also show in turn that at the intra-group level, different activity structures may affect people’s exposure levels, and the daily activity structure does have a certain impact on people’s final exposure results.

## 5. Conclusions

By examining the relationship between people’s activity structure and their PM_2__.__5_ exposure levels, this paper provides a new perspective for addressing when, where and how individuals interact with places and how their daily activity structures affect their exposure levels. The association between people’s daily activity structure and exposure to PM_2__.__5_ is quantified using the mobile-phone GPS location dataset of a weekday in Wuhan. First, two types of distance matrices between individuals of the sample population are constructed (i.e., the activity structure distance matrix and PM_2__.__5_ exposure distance matrix). Pearson correlation analysis and linear regression model were conducted on the two matrices to reveal the correlation. 

The Pearson correlation coefficient between the distance of activity structure and the distance of PM_2__.__5_ exposure is 0.78 (*p* < 0.01). The results of the linear regression model show a positive relationship between the distance of PM_2__.__5_ exposure and the distance of activity structure vector (with a slope of 7.34 and an R^2^ of 0.60). This indicates that there is a strong linear relationship between individual activity structure and PM_2__.__5_ exposure. The smaller the distance of activity structure between two individuals, the closer are the PM_2__.__5_ exposures between them and vice versa. In other words, people with similar activity structures tend to have similar exposure levels to PM_2__.__5_, while people with greatly different activity structures tend to have large differences in their exposure levels to PM_2__.__5_. 

We used the K-means algorithm to cluster the sample individuals into four distinctive groups (factory workers, office workers, adventurers and education-related workers) and compared the inter-group difference and intra-group difference in the relationship between activity structure and PM_2__.__5_ exposure. 

At the inter-group level, an ANOVA test was conducted and the F-value (4596.97) is much larger than the critical value of F (2.6056) when the level of significance is 0.05, which means that the mean PM_2__.__5_ exposure levels of the four activity structure groups differ significantly from each other. Then, the post-hoc procedure of the Scheffé test was performed and the F-values between all pairs of activity structure groups are greater than F-Critical (7.8168) except for the pair of adventures and education-related groups. The results indicate that different activity structure groups do experience different levels of PM_2__.__5_ exposure. Among the four groups, factory workers tend to have the highest exposures; office workers have relatively high exposures; the education-related group tends to have low exposure; while the adventurers have the lowest exposures among the four groups. Then, the relationship between activity structure distance and PM_2__.__5_ exposure distance among the four groups is examined. The order of the distance of PM_2__.__5_ exposure between activity structure groups is consistent with the order of the distance of activity structure between activity structure groups. The results show that, at the activity structure group level, the relationship between the two variables (activity structure and PM_2__.__5_ exposure) is also true. The smaller the distance between the activity structures of two activity structure groups, the closer is the distance between their PM_2__.__5_ exposures and vice versa. 

At the intra-group level, differences in the detailed characteristics of activity structure for people at different exposure levels within each group are also observed. For factory workers, people with higher exposure levels have longer work hours and the types of recreational activities are more monotonous than people with lower exposure levels. For adventures, people with high exposure levels visit leisure and entertainment facilities, shopping-related places and restaurants more, the main travel mode is the private car and the use of green spaces is very low. However, people with low exposure levels are more inclined to visit tourist places and travel by public transportation, and the levels of entertainment and shopping activities are relatively low. For education-related, people with low exposure levels have a higher willingness to go out than those with medium-to-high exposures, and the places they visited are diverse. These results indicate that individuals with different activity structures tend to experience different exposures, and human activity structure has a significant influence on people’s exposure to environmental risk factors.

The findings of this study emphasize the need for public health interventions and urban planning initiatives to mitigate social disparities in exposure to air pollution and alleviate health disparities across socioeconomic groups. More attention needs to be paid to the groups with higher exposure risk. Also, changing when and where to undertake activities can change the exposure level as well, because specific activity patterns are associated with specific exposure levels. This work also indicates that interdisciplinary research connecting researchers in health geography, computer sciences and human dynamics can generate useful information for a better understanding of the interaction between human behavior and environmental factors. 

There are several limitations in this study. First, it is recognized that indoor air pollution levels may be different from outdoor air pollution levels, we do not have data on indoor air pollution levels. As a result, we assume in this study that air pollution levels in different microenvironments (e.g., indoors) are the same as the outdoor levels estimated by Kriging interpolation due to data limitations. Second, there are other limitations associated with the strict correspondence between PM_2__.__5_ concentrations and users’ activity in indoor and outdoor spatio-temporal semantics. Due to the accuracy of the large-scale cell phone GPS dataset, it is still challenging to distinguish indoor and outdoor activities based on GPS trajectory data. In future studies, the trajectories of indoor and outdoor should be separated through the analysis of the spatio-temporal activity patterns of the trajectories, and the indoor air pollution measurement method should be used to assist in establishing a suitable correspondence between PM_2__.__5_ concentrations and city-wide activity structures, which provides semantically consistent data for the quantitative study of the relationship between individual’s activity structure and PM_2__.__5_ exposure level at the urban scale.

## Figures and Tables

**Table 1:** The corresponding relationship between the definition of social character for each activity structure group and POI category.

#	Main POI Category	Activity Structure Characteristic	Social Character of Group
1	Industrial park	Factory-centered	Factory workers
2	Company	Office-centered	Office workers
3	Home	Home-centered	Stay-at-home
4	Tourist Place	Outgoing-centered	Adventurers
5	Education	Education-centered	Education-related

**Table 2:** Pearson’s correlation coefficient for the whole sample and each activity structure group.

Group	Pearson’s Correlation Coefficient *r*	N
*G_s_*	0.77 **	12,362
*G_f_*	0.85 **	3175
*G_o_*	0.73 **	2931
*G_a_*	0.76 **	2184
*G_e_*	0.81 **	4072

** *p* < 0.01. *G_s_* represents the whole sample excluding the group of stay-at-home, *G_f_* represents the group of factory workers, *G_o_* represents the group of office workers, *G_a_* represents the group of adventurers, *G_e_* represents the group of education-related. N represents the number of samples for each group.

**Table 3:** The results of the linear regression model for the whole sample and each activity structure group.

Group	R	R Square	Std. Deviation
*G_s_*	0.77	0.60	0.64
*G_f_*	0.85	0.72	0.54
*G_o_*	0.73	0.53	0.61
*G_a_*	0.76	0.58	0.68
*G_e_*	0.81	0.64	0.59

Independent variable: activity structure distance, dependent variable: PM_2__.__5_ exposure distance, Std. deviation: Std. Error of the estimates.

**Table 4:** The results of the ANOVA test among the four activity groups.

	Sum of Squares	df	Mean Square	F	Sig.
Between Groups	136,842.25	3	45,614	4956.966	2.6056
Within Groups	113,721.92	12,358	9.20		
Total	250,561.17	12,362			

Sum of squares represents the total amount of dispersion, df is the degrees of freedom, and mean square represents the variance among sample means.

**Table 5:** The results of the Scheffé test among the four activity groups.

Activity Structure Group Pair	F Value	F-Critical
(factory workers, office workers)	7.9751	7.8168
(factory workers, adventures)	12.3479	7.8168
(factory workers, education-related)	11.4524	7.8168
(office workers, adventures)	9.7514	7.8168
(office workers, education-related)	8.5893	7.8168
(adventures, education-related)	4.6166	7.8168

## Data Availability

The data and code that support the findings of this study are available in the OneDrive repository with the identifier(s) at the private link https://figshare.com/s/bc816583a551eca1ca4b accessed on 23 April 2021.

## Appendix A. The Pseudo Code for the K-Means Algorithm

**Algorithm A1:** K-means algorithm based on cosine similarity
**Input**: Sample Sets $S=\left\{S_{1},S_{2},\ldots ,,S_{n}\right\}$;Cluster number K
**Output**: Cluster after division $C=\left\{C_{1},C_{2},\ldots ,,C_{n}\right\}$
1: select k samples from S random as the initial mean vector$\;\left\{\mu_{1},\mu_{2},\ldots ,,\mu_{k}\right\}$
2: $C_{i}=\emptyset \;$
3: **for** j=1 →m **do**
4: Calculate the distance between $S_{i}$ and $\;d_{ij}$
5: Determine the cluster label of $\lambda_{i}$ by$\;\lambda_{i}=argmin_{i\epsilon \left\{1,2,\ldots ,k\right\}}\;d_{ij}$
6: Divide $x_{j}$ into corresponding cluster $C_{\lambda_{j}}$: $C_{\lambda_{j}}=C_{\lambda_{j}}\cup \{\}x_{j}$
7: **end for**
8: **for** i=1 →k do
9: Calculate the new mean vector: $\mu_{i}^{'}=\frac{1}{\left|C_{i}\right|}\sum_{x\epsilon C_{i}}x$
10: **if** *u_i_**’ ≠* *u_i_* **then**
11: Update*u* with *u_i_’*
12: **Else**
13: Keep μ unchanged
14: **end if**
15: **end for**
16: **until** None of mean vectors will updated
